# Bicycle and Electric Scooter Fatalities: A Systematic Review of Micromobility-Related Deaths

**DOI:** 10.7759/cureus.111017

**Published:** 2026-06-17

**Authors:** Athina Tousia, Nikolaos Kalogrias, Konstantinos Katsos, Dimitrios Vlachodimitropoulos, Chara Spiliopoulou, Emmanouil I Sakelliadis

**Affiliations:** 1 Department of Forensic Medicine and Toxicology, National and Kapodistrian University of Athens, School of Medicine, Athens, GRC; 2 Forensic Service of Athens, Ministry of Justice, Athens, GRC

**Keywords:** bicycle accidents, e-scooter accidents, helmet use, injury epidemiology, road safety, toxicology, two-wheeler fatalities

## Abstract

Two-wheelers, including bicycles and electric scooters (e-scooters), account for a significant portion of road traffic fatalities. Although there are several measures, including alcohol and helmet legislation, mortality rates remain high, specifically in low- and middle-income countries. This systematic review aims to identify and synthesize factors associated with fatal outcomes in bicycle and e-scooter collisions.

We conducted a systematic search across PubMed, Scopus, and Web of Science from 2014 to April 2026. The included studies were observational (cohort, case-control, and cross-sectional) and examined fatal bicycle and e-scooter crashes. Data extraction centered on helmet use, substance use involvement, injury patterns, and demographic variables. Study quality was assessed using the Joanna Briggs Institute (JBI) methodology for systematic reviews. Due to substantial heterogeneity in study design, populations, vehicle type, outcome definitions, and reported variables, a meta-analysis was not performed. Instead, findings were synthesized using a narrative approach.

A total of 26 studies were included, including data from across the globe. Age, as well as sex distribution, was reported in 23 studies. Helmet use was reported in 14 studies. Injury patterns were described in 18 studies. Toxicological findings were reported in 14 studies. The lack of helmet use appeared to be linked to mortality. Alcohol use, although not frequently analyzed, was also significantly correlated with mortality. Elderly victims of male sex appear to demonstrate the highest mortality numbers.

Fatalities from bicycle and e-scooter collisions result from several commonly modifiable behavioral and environmental factors. The establishment of intervention strategies, including helmet laws, substance abuse prevention, infrastructure improvements, and age- and gender-targeted education, is crucial to reducing mortality. Enhanced prehospital care and injury surveillance systems are also mandatory to improve survival outcomes.

## Introduction and background

The growing use of micromobility options (a range of small, lightweight vehicles), such as bicycles and electric scooters (e-scooters), has significantly affected urban mobility in recent years. These means offer flexibility, economy, and environmental benefits [1]; however, they are associated with an increased risk of injury, especially in high-traffic load areas [2]. Traffic accidents involving bicycle and e-scooter users are a major public health issue, since they often result in serious injuries as well as fatalities [3]. Bicycles and e-scooters were examined together because both are vulnerable, unprotected road users exposed to direct impact forces with limited external protection and operating in similar urban traffic environments. However, findings were interpreted separately where possible because the two vehicle types differ in speed, user behavior, infrastructure use, and injury mechanisms. Head and brain injuries are particularly serious and are the leading cause of death in this type of accident [4,5]. At the same time, factors such as not wearing a helmet, alcohol or other substance use, and the circumstances of the accident (night riding, urban environment) appear to significantly influence the outcome [6-8]. Although the literature on bicycles is extensive, data on e-scooters are rather limited, reflecting their rapid but relatively recent integration into the modern urban environment [9]. Furthermore, the differences in injury patterns and risk factors between these two user groups have not been fully interpreted [10]. The purpose of this systematic review is to investigate fatal injuries and associated risk factors in traffic accidents involving bicycle and e-scooter users. Although several studies have examined micromobility-related injuries, fewer reviews have focused specifically on fatal cases and on forensic variables such as cause of death, injury patterns, toxicological findings, and helmet use. Therefore, this review aimed to synthesize fatal bicycle and e-scooter cases with emphasis on demographic, toxicological, traumatic, and forensic characteristics.

We conducted a systematic literature review using the Preferred Reporting Items for Systematic Reviews and Meta-Analyses (PRISMA) 2020 Guidelines [11]. This review was not prospectively registered in the International Prospective Register of Systematic Reviews (PROSPERO). This was acknowledged as a limitation. However, the methodology, eligibility criteria, search strategy, study selection, and synthesis approach are reported in detail to improve transparency and reproducibility.

We searched databases including PubMed, Scopus, and Web of Science. Searches were conducted from January 2026 to April 2026, with the final search completed in April 2026. Grey literature, government traffic reports, unpublished forensic databases, and surveillance systems were not systematically searched. This was acknowledged as a limitation because relevant fatality data may be available outside peer-reviewed literature.

Search words included: “Bicycle”, “e-scooter”, “Fatal”, “Collision”, “Fatal Injury”, “Cyclist”. Only studies published between 2014 and April 2026 were eligible for inclusion. Older references were used only for background or methodological context and were not included in the systematic synthesis.

The search strategy was expanded beyond isolated keywords and included synonyms and related terms for bicycles, e-scooters, micromobility, fatal outcomes, road traffic crashes, forensic investigation, helmet use, injury patterns, and toxicological findings. Boolean operators were used, and the syntax was adapted for each database.

For PubMed, the search was performed using title/abstract terms for bicycles, cyclists, e-scooters, fatal outcomes, and road traffic crashes. The final PubMed search string was: ((bicycle[Title/Abstract] OR cyclist[Title/Abstract] OR "e-scooter"[Title/Abstract] OR "electric scooter"[Title/Abstract]) AND (fatalit*[Title/Abstract] OR "fatal injur*"[Title/Abstract]) AND (crash*[Title/Abstract] OR collision*[Title/Abstract] OR "road traffic"[Title/Abstract])) AND ("2014/01/01"[Date - Publication] : "2026/04/30"[Date - Publication])**

In Scopus, the search strategy was adapted using the TITLE-ABS-KEY field and combined terms related to bicycles, cyclists, scooters/e-scooters, fatal outcomes, and crash context. The final search string was: TITLE-ABS-KEY ( bicycle OR cyclist OR cycling OR scooter* OR "electric scooter" OR "e-scooter" ) AND TITLE-ABS-KEY ( fatalit* OR "fatal injury" OR "fatal injuries" ) AND TITLE-ABS-KEY ( crash* OR collision* OR "road traffic" ) AND PUBYEAR > 2013 AND PUBYEAR < 2027**.

In Web of Science, the search strategy combined terms related to the vehicle type, fatal outcome, and crash context. The final search string was: **(bicycle* OR scooter*) AND fatal* AND crash***. Filters were applied for publication year from 2014 to April 2026, English language, and article document type.

The Population, Exposure, Comparator, and Outcome (PECO) framework was used for the inclusion criteria [12]. We removed duplicates and screened titles/abstracts using Rayyan (Qatar Computing Research Institute, Doha, Qatar) [13]. The screening was performed by two reviewers. Titles and abstracts were screened independently by two reviewers according to the predefined eligibility criteria. Records that clearly did not meet the inclusion criteria were excluded. 

Full-text articles were then assessed for eligibility. Full-text eligibility was initially assessed by one reviewer and subsequently checked by a second reviewer for excluded or uncertain articles. Inter-reviewer agreement was not formally assessed using Cohen’s kappa statistic. Disagreements between reviewers during title and abstract screening were resolved through discussion and consensus. The absence of a formal agreement statistic is acknowledged as a limitation. The study selection process was summarized using a PRISMA flow diagram. Reasons for full-text exclusion were recorded and reported.

The eligibility criteria were defined according to the PECO framework. The population of interest comprised cyclists and e-scooter riders involved in road traffic accidents, irrespective of age or sex. The exposure was defined as helmet use, alcohol/drug involvement, age, sex, crash type, vehicle type, and road environment. Comparator: where available, helmeted vs. non-helmeted, alcohol-positive vs. alcohol-negative, bicycle vs. e-scooter. The primary outcomes assessed were fatality and mortality. Studies were included if they were primary research articles published in English between 2014 and 2026. Exclusion criteria encompassed non-English literature, case reports, review articles, conference proceedings not published as full-text peer-reviewed journal articles, abstracts, citations, theses, and unverified or unsubstantiated reports from press or news media sources.

We used a pre-defined data extraction form that captured authorship, country, design of the study, number of cases (fatal), type of vehicle, types of injuries, toxicology results, use or non-use of helmets, and demographic characteristics. Finally, we grouped the findings by themes (demographics, injuries, and toxicology results). 

This systematic review was conducted in accordance with the Joanna Briggs Institute (JBI) methodology for systematic reviews (Appendix A) [14]. This study aims to systematically review the literature to identify and synthesize the factors contributing to mortality in two-wheeled accidents. The research questions investigated included risk factors (demographic and medical) for mortality in traffic accidents regarding bicycle and e-scooter collisions, the most common injury patterns found in such incidents, and the role of helmets in survival outcomes. Studies rated as “good” generally had clearly defined populations, valid outcome measures, and appropriate statistical analysis. Studies rated as “moderate” commonly lacked complete reporting of confounders, helmet status, toxicology, or standardized definitions of fatality. The risk-of-bias assessment was used to guide interpretation of the findings. Conclusions drawn from studies with incomplete exposure data, small sample sizes, or unclear definitions of mortality were interpreted cautiously.

Due to substantial heterogeneity in study design, population characteristics, vehicle type, outcome definitions, exposure variables, and reporting of effect estimates, a meta-analysis was not performed. Instead, findings were synthesized narratively. The narrative synthesis grouped findings by study characteristics, vehicle type, demographics, helmet use, toxicological findings, injury patterns, cause of death, and forensic relevance. Where possible, results were summarized using counts and proportions across the included studies, such as the number of studies reporting age, sex, helmet use, toxicology, and injury patterns. Because of heterogeneity and inconsistent reporting, pooled effect estimates, odds ratios, relative risks, and formal subgroup analyses were not calculated. Therefore, causal conclusions were avoided, and the findings were interpreted as descriptive associations rather than as definitive determinants of mortality.

## Review

Study selection

**Figure 1:**
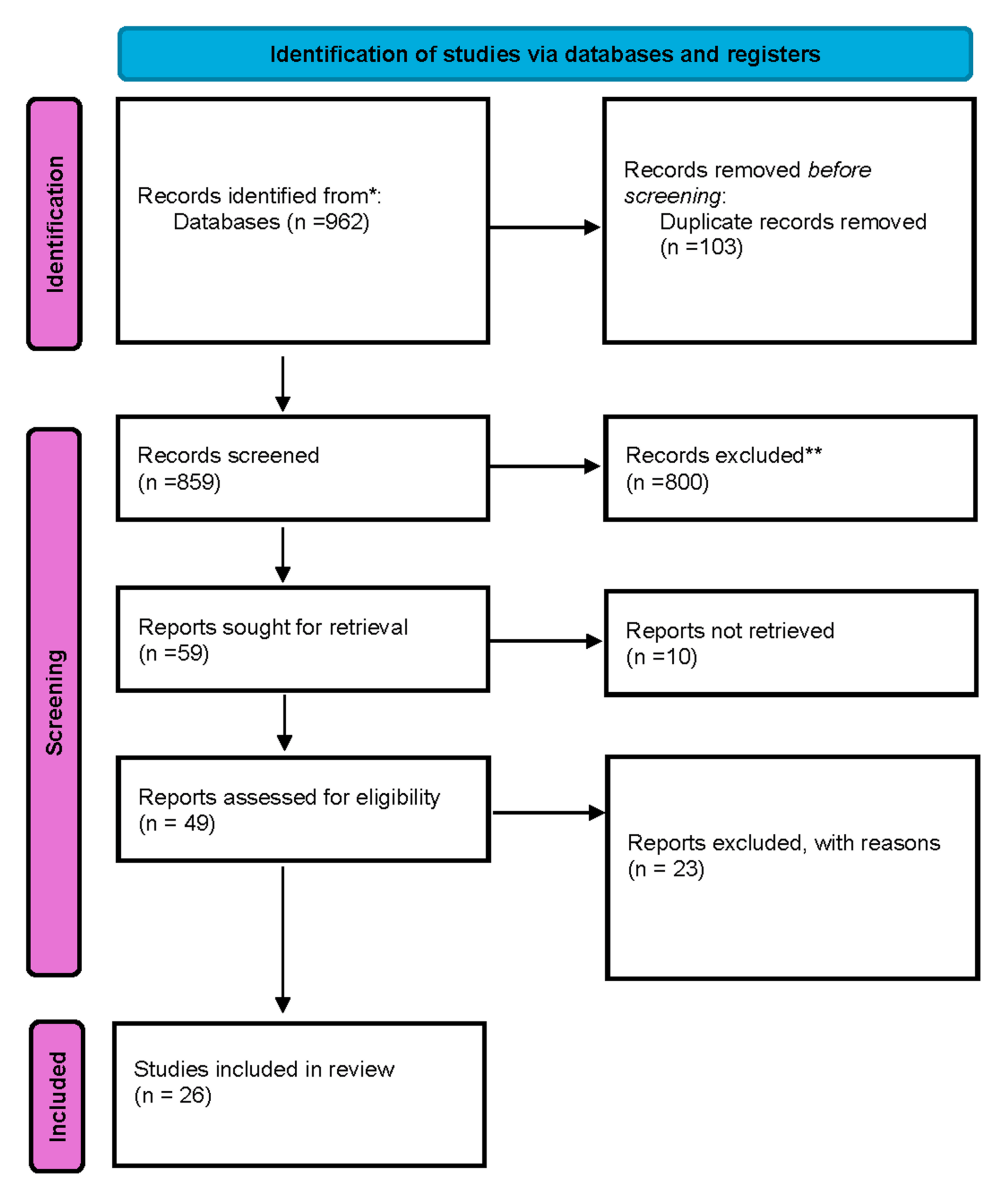
A PRISMA flowchart outlining the study selection process

We conducted a systematic literature search across databases and identified publications on road traffic accidents involving bicycles and e-scooters. After duplicate records were removed, the remaining articles underwent an initial screening of titles and abstracts. Studies unrelated to fatalities, did not involve bicycles or e-scooters, or did not provide sufficient data, were excluded at this stage. Subsequently, a full-text assessment of potentially eligible publications was performed. Based on the pre-specified inclusion and exclusion criteria, 26 studies met the inclusion criteria and were included in the final analysis of this systematic review. The study selection process is presented in the PRISMA flowchart (Figure 1).

General characteristics of the included studies

The studies displayed considerable heterogeneity in terms of design, sample size, and geographical distribution, originating from countries from all regions, including Europe, Asia, America, and Africa. The majority were retrospective epidemiological studies, analyses of national traffic accident databases, or hospital and forensic case series focused on factors contributing to mortality, including demographics, helmet use, substance use, and injury patterns. In several cases, substantial national traffic accident registries were used, including thousands of cases, while other studies were based on smaller case series from hospitals or forensic services. The studies focused on cyclists and e-scooter users. Full-text articles excluded, with reasons (n = 23): nonfatal outcomes only (n = 20), wrong population/vehicle type (n = 1), review/editorial/commentary (n = 1), full text unavailable (n = 1). Included studies are shown in Table 1. 

**Table 1:** Studies included in the systematic review DSP: disease surveillance points; BAL: blood alcohol level; RTC: road traffic crash; TBI: traumatic brain injury; DUI: driving under the influence; LSD: lysergic acid diethylamide

Study Title	Authors	Year	Country	Sample Size	Age (Years)	Sex	Helmet Use	Toxicology	Injuries	Main Findings
Bicyclist mortality between 2006 and 2010 in China [15]	Zhou M, Hu G, Wang L, et al.	2014	China	73 million (total DSP coverage), 5560 bicyclist deaths	Mortality was highest in older adults; ≥60 years had the highest risk	Males had 2.36-fold higher mortality than females	Not reported	Not reported	Not reported	Between 2006 and 2010, the mortality rate for bicyclists increased from 1.1 to 1.6 per 100,000 population
Comparison of the injury severity and medical history of disease-related versus trauma-related bicyclist fatalities [16]	Hitosugi M, Koseki T, Miyama G, Furukawa S, Morita S.	2015	Japan	55	Reported	Mostly male	Low (two victims)	16 victims positive for alcohol	Yes, brain or cervical spinal injuries	39 victims (70.9%) died of accident-related trauma
Factors affecting bicycle fatal and serious injury crashes in Victoria [17]	Bahrololoom S, Moridpour S, Tay R	2016	Australia	11,336, 120 bicycle fatalities	26 to 45 years old (48.0%)	Not reported	76.7% wore a helmet	Not reported	Not reported	Age and helmet use were significant variables affecting crash severity
Alcohol use by urban bicyclists is associated with more severe injury [18]	Sethi M, Heyer J, Wall S, et al.	2016	USA	689	84.9% were male with a mean age of 35.2 years.	Mostly male	Yes, less likely among alcohol users	Yes; BAL >0.01 g/dL considered positive	More severe in the alcohol group (head, face, abdomen, spine)	Alcohol use is linked to more severe trauma and higher mortality
Recent trends in cyclist fatalities in Australia [19]	Boufous S, Olivier J	2016	Australia	All national fatalities 1991–2013	Mean age 45.3 years in single-vehicle fatalities vs. 36.2 years in multivehicle fatalities	Not reported	Not reported	Not reported	Not reported	The average age of single vehicle crash fatalities was 45.3 years, while in multivehicle crashes was lower (36.2 years)
Bicycle fatalities: Trends in crashes with and without motor vehicles in The Netherlands [20]	Schepers P, Stipdonk H, Methorst R, Olivier J	2017	Netherlands	Years 1996-2019 (191.9 average annual fatalities)	Elevated risk of fatality for ages 75+	Not reported	Not reported	Not reported	Not reported by type	The elderly have an elevated risk
Fatal cyclist crashes in Australia [21]	O’Hern S, Oxley J	2018	Australia	336 fatalities	Mostly 35-64 years	Majority male	Helmet in 62%	Alcohol 14.6%, drugs 17.6%	Multiple injuries, head trauma	Vehicle collisions major mechanism
Features of fatal injuries in older cyclists in vehicle–bicycle accidents in Japan [22]	Matsui Y, Oikawa S, Hitosugi M	2018	Japan	2009-2013	Focus on ≥75 years; this group accounted for 43%–44% of fatalities	In the 75+ group, more male than female fatalities	A helmet is a protective factor	Not reported	Predominantly head injuries; fatal hip injuries are also important in 75+ cyclists, especially women	Cyclists aged 75 years and over had significantly higher percentages of hip injuries (hood-type vehicles)
Differences in severity of injuries between motorcyclist and bicyclist fatalities in single vehicle collisions [23]	Takeda A et al.	2020	Japan	14 bicyclist fatalities	Mean age not reported separately for bicyclists; overall cohort mean 62.3 years	23 male, two female	None wore a helmet	Alcohol was present in 52%	Lower overall injury severity than motorcyclists, but more severe facial injuries; neck injuries were notable, and alcohol-positive bicyclists had more severe neck and upper-extremity injuries	Alcohol associated with more severe injuries
Bicycle safety in Bogotá: A seven-year analysis of bicyclists’ collisions and fatalities [24]	Carvajal GA et al.	2020	Colombia	Fatal collision dataset	Not reported	Fatal bicycling collision rates per bicyclists’ population remained stable for females and decreased 53 % for males	Not reported	Not reported	Severe trauma	Fatal collisions associated with heavy vehicles
Childhood cycling fatalities in South Australia before and after the introduction of helmet legislation [25]	O’Donovan S, van den Heuvel C, Baldock M, Byard RW	2020	Australia	36 deaths in 1982–1991, 12 deaths in 1992–2001	Four to 14 years; mean 10.2 years	Male:female = 5:1	No	Not reported	Not reported for the study cases. The paper states generally that the main cause of death in cycling crashes remains cranial trauma	Need for a child-specific road safety policy
Bicycling-related mortality in Ecuador [26]	Cordovez MA, Arce C, Maldonado A	2021	Ecuador	300	The mortality rate increased with age	91% of the victims were men	Not reported	Not reported	Head (mostly inferred), extremities	91% of the victims were men, while 9% were women
Comparison of Injuries Associated With Electric Scooters, Motorbikes, and Bicycles in France [27]	James A et al.	2023	France	A total of 5,233 patients	Median 33 years (IQR 25–46)	83.7% male	22.5% wore helmets	36.7% BAL above legal threshold	TBI was the most common cause of death	25.9% had severe TBI; e-scooter RTC mortality was 9.2%; injuries as severe as those from bicycles and motorbikes
Mortality due to traffic accidents in Colombia [28]	Garcia G, Santos J, Martinez F	2023	Colombia	8,023 cyclists (24% fatal)	Mostly <25 years (30.3%)	About 13 male deaths for every one female death	Not reported	Not reported	Not reported	The younger age group among cyclists is an important predictive factor
On a collision course: fatal motorcycle and bicycle accidents of adolescents in Finland from 2008 to 2019 [29]	Unkuri et al.	2024	Finland	147 fatalities (20 bicycle, 50 moped, and 77 motorcycle riders)	Mean 15.5 years	55% male	90% had no helmet	0 DUI cases	Head is the most common	One-fifth of all-cause mortality among 15-year-olds was due to two-wheeled vehicles accidents
Protective effect of helmet use on mortality in bicycle crashes [30]	Yeon J, Kim S, Kong J, Park G	2024	South Korea	76,983 bicycle crashes (282 fatalities)	The majority of fatalities were 60–79 years old (58.6%)	88% male	Low (11.1% overall, 3.9% among deaths)	Alcohol use is associated with mortality	Head injuries are common, with TBI in 41.5% of fatalities	Key mortality factors include time of injury, alcohol use, crashes on national highways, no helmet use, and collisions with automobiles.
Fatal traffic accidents involving electric scooters in Poland in 2019–2023 [31]	Strnad S et al.	2024	Poland	Nine cases	Mean 40.3 years	77.8% male	1/9 helmeted	Alcohol in 2/9, one case with LSD and citalopram	Craniocerebral injury was the most common cause of death	High proportion of head trauma; 43.8% deaths were immediate, the most common cause of death was craniocerebral trauma
Cycle fatalities in Delhi and their risk factors [32]	Agrawal S et al.	2024	India	167 fatal crashes	Most fatalities were 30–59 years old	98.4% male, 1.6% female	Not reported	Not reported	Not reported	Road design and heavy vehicles increase risk
Trends in and risk factors for bicycle-related mortality in Japan [33]	Tanaka S et al.	2025	Japan	National mortality data	60% of deaths were in riders >70	Fatality-only sex distribution not reported	Not reported	Fatality-only not reported; 8.41% drinking in injury database	Predominantly head injuries (54.06% in the bicycle injury database)	Mortality risk rises steeply with age
Evaluating the impact of cycle helmet use on severe traumatic brain injury and death in a national cohort of over 11000 pedal cyclists [34]	Dodds et al.	2019	UK	11000 Cyclists involved in accidents	Adults ≥16 years; fatality-only age not reported	84.7% male overall. Fatality-only sex was not reported.	Helmet use is associated with lower 30-day mortality	2.1% in helmeted cyclists and 15.6% in non-helmeted cyclists. Fatality-only toxicology was not reported	Severe TBI and facial injury were less common in helmeted cyclists	Helmet use is associated with reduced rates of fatality and brain injury
Severe Injuries in E-Scooter Accidents: An Evaluation of Data From the TraumaRegister DGU [35]	Hartz et al.	2025	Germany	538 cases of severely injured e-scooter riders, 26 fatal	Mean 44.3 years; fatality-only age not reported	78.4% male; fatality-only sex not reported	Not reported	34.9% alcohol-positive/under the influence; fatality-only not reported	Head injuries predominant	High rate of severe injuries, especially head trauma
Contribution of exposure, risk of crash and fatality to explain age- and sex-related differences in traffic-related cyclist mortality rates [36]	Martínez-Ruiz et al.	2015	Spain	Large dataset	Five to 79 years; mortality increased with age	Higher mortality in males in every age group	Not reported	Not reported for the study cases.	Not reported for the study cases.	Age and sex significantly influence mortality
Circumstances and causes of fatal cycling crashes in the Czech Republic [37]	Bíl et al.	2016	Czech Republic	129 cyclist fatalities	The most common age group is 51–60 years	103 male, 26 female	Five helmeted	Alcohol common; 34/93 tested had BAC >1.00 g/kg	Intracranial injuries were the leading cause of death (52.7%)	Crash circumstances key factor
Trends in bicycle-related injuries, hospital admissions, and deaths in the USA 1997-2013 [38]	Fergus et al.	2019	USA	Large dataset	Not reported for fatalities only	Male predominance	Not reported for fatalities only	Not reported for fatalities only	Severe injuries	Increasing injury and death trends
Cyclists fatalities: Forensic remarks regarding 335 cases [39]	Piras et al.	2016	Italy	335	Mean age 58 years; most fatalities in older adults	77.62% male	0 helmeted	14/19 tested were positive, alcohol and cannabinoids were detected	The head was the most frequently involved region in lethal injuries (65.37%)	Head injuries most common cause of death
Postmortem injury quantification for the fatally injured cyclists in the Osijek-Baranja county over a 21-year period [40]	Marinović et al.	2021	Croatia	125 cyclist fatalities	The majority were older than 45 years (80.8%).	80% male	78.4% were not wearing a helmet	DUI of alcohol is an important prevention target.	The majority had severe head and brain injuries	Injury quantification confirms severity patterns

Table 1 demonstrates substantial heterogeneity in sample size, study design, and reported variables. Some studies were national registry analyses, whereas others were small forensic case series, limiting direct comparison. Age and sex were reported in 23 out of 26 studies, helmet use in 14 out of 26 studies, toxicological findings in 14 out of 26 studies, and injury patterns in 18 out of 26 studies. This heterogeneity limited direct comparison and precluded meta-analysis.

Demographic characteristics

Among the 26 included studies, sex distribution was reported in 23 studies, and these generally showed a predominance of male victims. Age was also examined in 23 of 26 studies. Almost all studies reported that victims of such fatalities were predominantly male. Age was examined in most studies. The age groups most frequently affected were children and older adults. This underlines the presence of a two-spike distribution. 

Helmet use

Helmet use was reported in 14 of 26 studies. Helmet use was one of the most frequently examined factors in the included studies and was analyzed in most. Most findings indicated that not wearing a helmet is linked to a significantly increased risk of head injury and higher mortality. 

Injury patterns

Injury patterns were described in 18 of 26 studies. In terms of injuries, head trauma was noted as the most common cause of death across bicycle and e-scooter fatalities, especially in non-helmeted riders. Chest and limb injuries were also very common.

Toxicological findings

Toxicological findings were reported in 14 of 26 studies. According to most of the studies, alcohol is a significant risk factor for mortality (reported as a significant risk factor for traffic collisions and was associated with an increased likelihood of fatal outcome). Substance use, as well as CNS-affecting medicine, was rarely examined. 

Cyclists

When it comes to bicycles, alcohol use and non-use of helmets are significant mortality factors. The most affected age groups remain the same, with a prevalence in the elderly population, with cyclists aged 75 years and over having significantly higher percentages of hip injuries. On the contrary, children and adolescents were also affected. For e-scooters in particular, most publications are relatively recent and indicate a rising incidence of injuries in urban settings, especially in warmer climates. The male population was again the most affected, while helmet use is a protective factor. However, the available data on fatal cases remains limited compared to the literature on motorcycle road traffic accidents.

E-scooter users

For e-scooters in particular, most publications are relatively recent and indicate a rising incidence of injuries in urban settings, especially in warmer climates. The male population again seemed to be the most affected, while helmet use was a protective factor. However, the available data on fatal cases remains limited compared to the literature on motorcycle road traffic accidents.

Discussion

Fatal road traffic accidents involving bicycles and e-scooters are a major public health concern worldwide. After evaluating the literature on bicycle fatalities, we identified critical factors contributing to mortality in bicycle accidents among adults and children. The results confirm that drug/alcohol use [18] as well as the absence of helmet use, are contributing factors to fatal accidents [36]. Specifically, the absence of helmet use was highlighted as a contributing factor in several studies across multiple countries [37-43]. Craniocerebral injuries are considered the main cause of death in most cases, especially in children [25]. Furthermore, thoracic and hip injuries are mostly seen in the elderly. Alcohol use before the accident is considered an independent mortality contributor [33]. 

In terms of demographic data, there is a clear male predominance among victims of fatal bicycle and e-scooter crashes [43,44]. Age may also be relevant to fatality patterns, as older age is associated with increased mortality due to increased vulnerability to serious injuries, particularly in countries with an aging cyclist population [44,45]. In pediatric and adolescent populations, the data regarding such crashes also appear to account for a substantial proportion of mortality in some studies, highlighting the need for targeted prevention interventions [42]. 

The use of protective helmets is recognized as one of the most important modifiable risk factors for RTAs. Large-scale studies show that helmet use is associated with a significant reduction in mortality and severe head injury [25]. Conversely, non-helmet use is associated with a higher risk of fatalities, particularly in urban environments with higher traffic [46]. Furthermore, implementing helmet legislation has been shown to reduce mortality, particularly in younger populations [25]. However, in many countries, compliance remains low, highlighting the need to strengthen prevention policies.

The present systematic review underlined the role of alcohol and substance consumption as an important contributing factor to fatality since toxicological findings play a crucial role in understanding the circumstances of such accidents. The presence of alcohol has been associated with increased injury severity and higher mortality [18]. When it comes to substance use, although rarely analyzed in the literature, it surely has a major effect in collision circumstances with the reported substances, including cannabinoids and lysergic acid diethylamide (LSD) [39], while CNS-affecting medications such as antidepressants have also been reported [31]. Polydrug use further complicates interpretation because the independent contribution of alcohol, cannabis, stimulants, sedatives, or other substances to crash risk and fatal outcome cannot be reliably separated in most observational studies. From a forensic perspective, toxicological findings are critical in reconstructing the circumstances of the accident and assessing the victim's behavior prior to the crash.

In terms of injury patterns, traumatic brain injuries are the leading cause of death among bicycle and e-scooter users, as documented in forensic as well as epidemiological studies [29,40]. Additionally, thoracic and abdominal injuries are also very common, significantly contributing to the fatal outcome, especially in high-energy collisions and in urban environments with heavy traffic [18,22]. 

When it comes to e-scooters, the literature about fatalities is rather limited, although the number of cases has increased [35,47]. E-scooter users are among the most vulnerable road users, as the absence of a protective framework significantly increases the risk of serious or fatal injuries even in moderate-intensity conflicts. The literature suggests that e-scooters contribute disproportionately to overall road mortality in many countries, particularly in areas where they are a frequent mode of transport, on working days, and during the warm months [31]. Geographically, studies show variations in injury types related to speed, helmet use, vehicle type, and road infrastructure. Systematic documentation and analysis of injury patterns are crucial not just for understanding the mechanisms but also for establishing preventive and diagnostic tools. The comprehensive use of forensic evaluation, combined with epidemiological data, can contribute to improved survival outcomes. 

This review has several limitations. The studies about two-wheel accidents identified in this systematic review were principally primary studies (retrospective). The term mortality is used differently across studies (at 48 hours, during hospitalization, and immediately), which complicates comparisons of data. Furthermore, besides the usage of the JBI methodology for systematic reviews, bias cannot be eliminated. Additionally, because most studies are conducted in specific countries, the generalizability of the findings may be limited. Also, several studies use data from before 2010, which may make the information presented outdated. Finally, several studies didn’t report information regarding all the components (toxicology, types of injuries, etc.), leading to reduced accuracy. Papers not written in English were excluded from the study.

Limitations

This review has several limitations. Most studies are conducted in specific countries; the generalizability of the findings may be limited. Also, several studies didn’t report information on all components (toxicology, types of injuries, etc.), reducing accuracy. The use of only English-language studies may have introduced language restriction bias and led to the exclusion of relevant studies from countries with extensive micromobility use. Grey literature, government reports, surveillance databases, and unpublished forensic records were not systematically searched. This may have resulted in the underrepresentation of fatality data available outside peer-reviewed literature. Also, several studies use data from before 2010, which may make the information presented outdated. Full-text screening by a single reviewer was acknowledged as a limitation because it may increase selection bias. Furthermore, the absence of a formal kappa statistic is a methodological limitation. Mortality definitions varied across studies, including death at the scene, death within 24-48 hours, in-hospital mortality, and fatal cases identified through forensic or registry databases. Therefore, outcomes were not pooled and were interpreted according to each study’s definition. The included studies identified in this systematic review were principally primary studies (retrospective). Furthermore, besides the usage of the JBI methodology for systematic reviews, bias cannot be eliminated. A formal Grading of Recommendations Assessment, Development, and Evaluation (GRADE) certainty-of-evidence assessment was not performed because of heterogeneity in study design, outcomes, and reporting. This is acknowledged as a limitation.

## Conclusions

This systematic review highlights the global and persistent burden of micromobility fatalities. Two-wheeler road traffic accidents affect not only younger demographics but also the elderly, underscoring the need for preventive measures. Across the included studies, recurrent themes included limited helmet use, severe head injuries, and, in a number of reports, the contribution of alcohol or other toxicological factors. These findings highlight the multifactorial nature of such fatalities and underscore the need for targeted prevention strategies, including improved helmet compliance and stronger road safety interventions. The contribution of this review lies not in identifying entirely new risk factors but in synthesizing fatal micromobility cases with emphasis on forensic variables, including cause of death, injury patterns, toxicological findings, helmet use, and reporting gaps.

## Appendices

Appendix A 

**Table 2:** JBI critical appraisal of studies assessed with the analytical cross-sectional checklist Abbreviations: JBI, Joanna Briggs Institute; Q, checklist question. Each item was rated as Yes (Y), No (N), Unclear (U), or Not applicable (NA). JBI Analytical Cross-Sectional Q1: Were the criteria for inclusion in the sample clearly defined? Q2: Were the study subjects and the setting described in detail? Q3: Was the exposure measured in a valid and reliable way? Q4: Were objective, standard criteria used for measurement of the condition? Q5: Were confounding factors identified? Q6: Were strategies to deal with confounding factors stated? Q7: Were the outcomes measured in a valid and reliable way? Q8: Was appropriate statistical analysis used?

Study	Design	JBI tool	Q1	Q2	Q3	Q4	Q5	Q6	Q7	Q8	Overall appraisal
Zhou et al. [15]	Analytical cross-sectional	JBI Analytical Cross-Sectional	Y	Y	Y	Y	Y	Y	Y	Y	GOOD
Hitosugi et al. [16]	Analytical cross-sectional	JBI Analytical Cross-Sectional	Y	Y	Y	Y	N	N	Y	Y	MODERATE
Bahrololoom et al. [17]	Analytical cross-sectional	JBI Analytical Cross-Sectional	Y	Y	Y	Y	Y	Y	Y	Y	GOOD
Boufous and Olivier [19]	Analytical cross-sectional	JBI Analytical Cross-Sectional	Y	Y	Y	Y	N	N	Y	Y	MODERATE
Schepers et al. [20]	Analytical cross-sectional	JBI Analytical Cross-Sectional	Y	Y	Y	Y	Y	Y	Y	Y	GOOD
O’Hern and Oxley [21]	Analytical cross-sectional	JBI Analytical Cross-Sectional	Y	Y	Y	Y	N	N	Y	Y	MODERATE
Matsui et al. [22]	Analytical cross-sectional	JBI Analytical Cross-Sectional	Y	Y	Y	Y	N	N	Y	Y	MODERATE
Takeda et al. [23]	Analytical cross-sectional	JBI Analytical Cross-Sectional	Y	Y	Y	Y	N	N	Y	Y	MODERATE
Carvajal et al. [24]	Analytical cross-sectional	JBI Analytical Cross-Sectional	Y	Y	Y	Y	Y	Y	Y	Y	GOOD
O’Donovan et al. [25]	Analytical cross-sectional	JBI Analytical Cross-Sectional	Y	Y	Y	Y	N	N	Y	Y	MODERATE
Cordovez et al. [26]	Analytical cross-sectional	JBI Analytical Cross-Sectional	Y	Y	Y	Y	N	N	Y	Y	MODERATE
James et al. [27]	Analytical cross-sectional	JBI Analytical Cross-Sectional	Y	Y	Y	Y	Y	Y	Y	Y	GOOD
Montenegro-Martinez et al. [28]	Analytical cross-sectional	JBI Analytical Cross-Sectional	Y	Y	Y	Y	N	N	Y	Y	MODERATE
Unkuri et al. [29]	Analytical cross-sectional	JBI Analytical Cross-Sectional	Y	Y	Y	Y	N	N	Y	Y	MODERATE
Rzepczyk et al. [31]	Analytical cross-sectional	JBI Analytical Cross-Sectional	Y	Y	Y	Y	N	N	Y	Y	MODERATE
Agrawal et al. [32]	Analytical cross-sectional	JBI Analytical Cross-Sectional	Y	Y	Y	Y	Y	Y	Y	Y	GOOD
Tanaka et al. [33]	Analytical cross-sectional	JBI Analytical Cross-Sectional	Y	Y	Y	Y	Y	Y	Y	Y	GOOD
Hartz et al. [35]	Analytical cross-sectional	JBI Analytical Cross-Sectional	Y	Y	Y	Y	N	N	Y	Y	MODERATE
Martínez-Ruiz et al. [36]	Analytical cross-sectional	JBI Analytical Cross-Sectional	Y	Y	Y	Y	Y	Y	Y	Y	GOOD
Bil et al. [37]	Analytical cross-sectional	JBI Analytical Cross-Sectional	Υ	Υ	Υ	Υ	Ν	Ν	Υ	Υ	MODERATE
Fergus et al. [38]	Analytical cross-sectional	JBI Analytical Cross-Sectional	Y	Y	Y	Y	Y	Y	Y	Y	GOOD
Piras et al. [39]	Analytical cross-sectional	JBI Analytical Cross-Sectional	Y	Y	Y	Y	N	N	Y	Y	MODERATE
Marinović et al. [40]	Analytical cross-sectional	JBI Analytical Cross-Sectional	Y	Y	Y	Y	N	N	Y	Y	MODERATE

**Table 3:** JBI critical appraisal of studies assessed with the cohort checklist Abbreviations: JBI, Joanna Briggs Institute; Q, checklist question. Each item was rated as Yes (Y), No (N), Unclear (U), or Not applicable (NA). JBI Cohort Q1: Were the two groups similar and recruited from the same population? Q2: Were the exposures measured similarly to assign people to both exposed and unexposed groups? Q3: Was the exposure measured in a valid and reliable way? Q4: Were confounding factors identified? Q5: Were strategies to deal with confounding factors stated? Q6: Were the groups/participants free of the outcome at the start of the study? Q7: Were the outcomes measured in a valid and reliable way? Q8: Was the follow-up time reported and sufficient? Q9: Was follow-up complete, and if not, were reasons described? Q10: Were strategies to address incomplete follow-up utilized? Q11: Was appropriate statistical analysis used?

Study	Design	JBI tool	Q1	Q2	Q3	Q4	Q5	Q6	Q7	Q8	Q9	Q10	Q11	Overall appraisal
Sethi et al. [20]	Cohort	JBI Cohort	Y	Y	Y	Y	Y	Y	Y	U	U	U	Y	MODERATE
Dodds et al. [36]	Cohort	JBI Cohort	Y	Y	Y	Y	Y	Y	Y	Y	U	U	Y	GOOD

**Table 4:** JBI critical appraisal of studies assessed with the case-control checklist Abbreviations: JBI, Joanna Briggs Institute; Q, checklist question. Each item was rated as Yes (Y), No (N), Unclear (U), or Not applicable (NA). JBI case-control checklist items Q1: Were the groups comparable other than the presence of disease in cases or the absence of disease in controls? Q2: Were cases and controls matched appropriately? Q3: Were the same criteria used for identification of cases and controls? Q4: Was exposure measured in a standard, valid, and reliable way? Q5: Was exposure measured in the same way for cases and controls? Q6: Were confounding factors identified? Q7: Were strategies to deal with confounding factors stated? Q8: Were outcomes assessed in a standard, valid, and reliable way for cases and controls? Q9: Was the exposure period of interest long enough to be meaningful? Q10: Was appropriate statistical analysis used?

Study	Design	JBI tool	Q1	Q2	Q3	Q4	Q5	Q6	Q7	Q8	Q9	Q10	Overall appraisal
Yeon et al. [30]	Case-control	JBI Case-Control	Y	Y	Y	Y	Y	Y	Y	Y	U	Y	GOOD
